# WiBISS: a tool to estimate avoided lost revenue of African swine fever wild boar vaccination at municipality level

**DOI:** 10.3389/fvets.2025.1667173

**Published:** 2025-10-24

**Authors:** Pablo Ibáñez-Porras, Jaime Bosch, Francesco Feliziani, Carmen Maresca, Jose M. Sánchez-Vizcaíno, Irene Iglesias, Cecilia Aguilar-Vega, Marta Martínez-Avilés

**Affiliations:** ^1^Animal Health Research Center (CISA-INIA), Spanish National Research Council (CSIC), Valdeolmos, Madrid, Spain; ^2^Department of Animal Health, VISAVET Health Surveillance Centre, Complutense University of Madrid, Madrid, Spain; ^3^Istituto Zooprofilattico Sperimentale Umbria-Marche, “Togo Rosati”, Perugia, Italy

**Keywords:** disease control strategies, cost analysis, pig production, wildlife, ASF vaccine, rapidrisk assessment

## Abstract

This study introduces the WiBISS model, a simulation tool designed to assess the economic and epidemiological impact of a hypothetical African Swine Fever (ASF) vaccination in wild boar in Northern Italy. Using real ASF outbreak data from January 2022 to June 2024, the model evaluates how prompt vaccination could reduce disease spread and economic losses. WiBISS integrates three modules: vaccination simulation, restriction zone estimation, and economic impact analysis. The first two use custom-built cellular automata (CA) in Python and ArcGIS Pro, modeling each ASF case as a cell that can be in one of three states: unvaccinated, infected, or vaccinated. Weekly iterations over 2.5 years simulate ASF progression and vaccination impact based on localized interactions and a defined vaccination radius. Three vaccination scenarios were tested: (1) a non-vaccination baseline; (2) an “ideal” scenario with immediate, 100% vaccination; and (3) multiple “realistic” scenarios with an 8-week delay and varied vaccination rates (25–75%) and radii (10–50 km). The most effective realistic scenarios (e.g., 75% vaccination rate, 50 km radius) showed a total loss of €601,800, close to the ideal scenario. WiBISS prioritizes usability over epidemiological complexity, omitting detailed virus transmission modeling to enhance applicability in data-scarce regions. Unlike detailed stochastic models, WiBISS offers rapid, economically grounded insights to guide initial outbreak response and resource allocation. Although it does not include domestic pigs due to differing transmission dynamics, WiBISS lays a foundation for phased, integrated wildlife vaccination planning that balances economic feasibility with ecological realism.

## 1 Introduction

The north-west of Italy, a region known for its high-density pig production and renowned pork products, is threatened by the presence of African swine fever (ASF), a highly contagious and lethal hemorrhagic viral disease that affects domestic pigs and wild boar, leading to significant economic losses (1–3). The absence of an effective treatment (4), the lethal and highly contagious nature of ASF, and the stability of ASF virus in the environment (5, 6) necessitate stringent biosecurity measures. With wild boar acting as a primary driver for the virus spread and maintenance in Europe (7–10), controlling ASF within these populations has become a key focus for safeguarding the swine industry. However, controlling ASF in wild boar present significant challenges that complicate efforts to eradicate the disease. Wild boar populations are often dense and widely distributed, with the Alpine landscape of North-western Italy making wild boar difficult to track. Early detection and rapid response under such conditions are constrained. In addition, implementing control measures such as culling can face public resistance, and the social factor has complicated ASF control measures in Italy (11).

In this context, wild boar vaccination has emerged as a promising approach to curbing the spread of ASF (3). While research on ASF vaccines for wild boar is ongoing, and there is no commercial vaccine available at this time in Europe, we anticipate the economic advantages of their future use in wild boar. Oral baits were used to immunize wild boar in Europe against CSF since the 1990’s for more than 15 years, reducing the incidence in areas of high wild boar density, like Germany (12). The vaccination program was largely successful, with some regions declared free from disease after mass oral vaccination campaigns.

With the simulation of a computational modeling tool which we have named WiBISS (Wild Boar Immunization Simulation System), based on cellular automata (CA) modeling, we estimate the economic benefits of wild boar vaccination for pig producers in Northern Italy. CA models can represent the spatial dynamics of disease transmission and the local interactions between individuals in a population with a grid-based approach in which each element has a state representing the health status of an element belonging to a complex system (13). The CA model can allow assessing how vaccinating animals in one area can affect disease transmission in the surrounding areas. Additionally, we can assess the economic impact by including information on the expenditure of commodities with and without ASF. This way, the WiBISS would integrate epidemiological data and simulate the potential economic outcomes of vaccinating wild boar against ASF.

In this study we define losses as lost revenue associated with ASF regionalization measures. These losses derive from regionalization restrictions affecting municipalities, where farms experience export restrictions and market stigma that reduce demand, leading to price drops often below production costs.

Our goal is not to understand or predict the dynamics of ASF transmission, that occurs through multiple routes (14), but to use real data on ASF spread to simulate a hypothetical scenario in which a vaccine had been promptly administered. We aim to quantify the losses that could be avoided by the use of vaccination to offer a strong economic justification for implementing vaccination strategies. By quantifying the potential cost savings and disease mitigation effects of wild boar vaccination, WiBISS offers evidence-based insights that can inform cost–benefit analysis to influence the decision on whether or not to vaccinate based on epidemiological and economic reasons.

## 2 Materials and methods

### 2.1 Data and software

**Figure 1:**
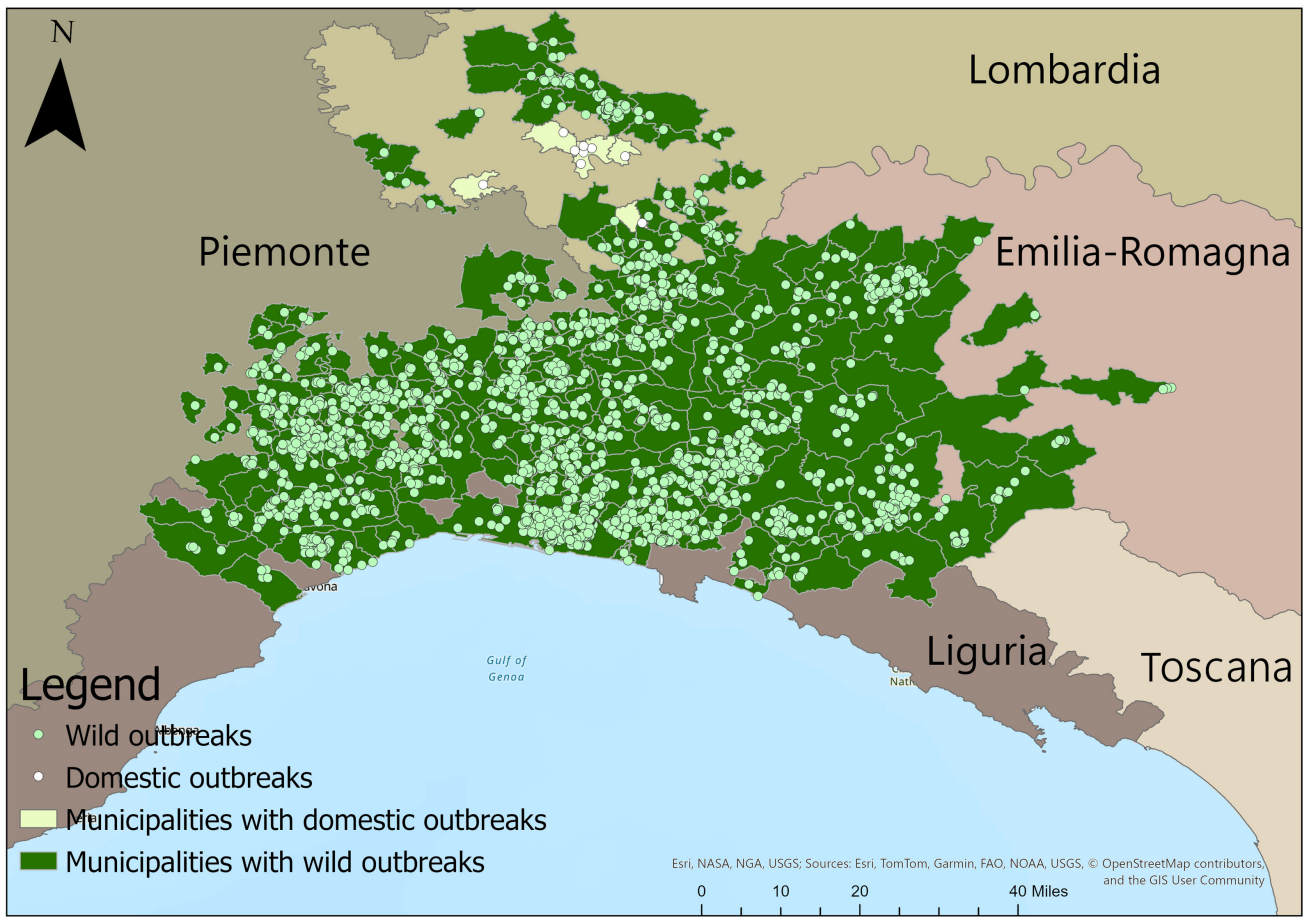
The figure illustrates a map showcasing the geographic locations of outbreak notifications from January 2022 to June 2024, with dark markers representing wild boar outbreaks and light markers indicating domestic pig outbreaks. Municipalities that have experienced wild boar outbreaks are highlighted in dark green, while those with domestic pig outbreaks are depicted in light green. Additionally, the affected regions are outlined to provide a comprehensive view of the distribution.

The study area comprises the ASF affected regions in Northern Italy (Emilia-Romagna, Liguria, Lombardy, and Piedmont) together with several municipalities in the Tuscany region, which fall within the neighborhood of municipalities with reported outbreaks within their boundaries. Figure 1 shows on a map the complete distribution of ASF outbreaks in northern Italy from January 2022 to June 2024.

Within the study area, we used WAHIS data (WOAH)^1^ for wild boar outbreak information (location, start date, species) and pig production data (location, type of production, species, exploitation, number of pigs per farm) from the VETINFO Italian informative system for animal health provided by the Istituto Zooprofilattico Sperimentale dell’ Umbria e delle Marche “Togo Rosati” (IZS-SUM) and the authorization of the Ministry of Health.

Data on vaccine characteristics (efficacy, safety) were obtained from research results from VACDIVA project.^2^ Since a commercial vaccine is still not available for ASF in Europe, we obtained data on field vaccination characteristics (effectiveness, immunity decay) by varying values based on CSF wild boar vaccination published research (12, 15) (see Supplementary material). Finally, we found monetary values for pigs, pork and pig products, within and outside the restriction zones in Italy, in the gray literature.^3^

The WiBISS model consists of three interconnected modules, each with a distinct function: wild boar vaccination simulation, restriction zone estimation based on ASF in wild boar, and economic impact analysis for domestic pig producers in restriction zones due to ASF outbreaks in wild boar. The first two modules are custom-built cellular automata (CA), tailored to their specific functions, while the third module serves to analyze the results. All mathematical algorithms have been formulated in Python 3.9.18 and charted in ArcGIS Pro 3.3 (©ESRI).

### 2.2 Vaccination simulation model

In the case of the CA model created to simulate a vaccination scenario, each cell of the CA represents an ASF case in wild boar notified between January 2022 (date of the first occurrence in the study area) up to June 2024, recreating a cellular space defined by the point-locations (longitude and latitude) of ASF cases, totaling 2,130 cells. This approach relies on the same principles of local grid-based neighbor interactions but is not constrained to a homogeneously spaced lattice.

The states of the cells were classified as “unvaccinated,” “infected,” or “vaccinated,” with “unvaccinated” referring to a cell that is neither infected nor vaccinated. The model is iterated weekly for the duration of the study period considered (2.5 years).

The evolution of each cell in a CA is defined by a set of transmission rules, which determine its next state as a function of its own state, the state of neighboring cells, and other factors. These rules are applied in discrete time steps and are detailed below. To complete the CA definition, a neighborhood must also be specified. In this model, we consider a cell’s neighborhood includes all other cells within a circular area centered on the cell, with a radius corresponding to the vaccination radius defined below.

#### 2.2.1 From unvaccinated to infected

Unvaccinated cells transition to the infected state when the iteration week matches their outbreak start date. Once a cell becomes infected, it remains in that state until the end of the simulation. Thus, transitions from infected to vaccinated and unvaccinated are not possible.

#### 2.2.2 From unvaccinated to vaccinated

To transition from unvaccinated to vaccinated, we considered several variables: the vaccination radius, the time before vaccination, vaccination effectiveness and immunity decay (see Supplementary material). Vaccination success in wild boar, according to Rossi et al. (12), relies on the season and year of deployment (natural food competing with feed stations and baits) and the delimitation of both the infection and the vaccination areas.

##### 2.2.2.1 Vaccination radius

First, an unvaccinated cell must be in proximity to an infected one. In terms of CA, a cell must be in the neighborhood of an infected cell to be also infected. In practice, the distance between cells is determined using the haversine formula, which relies on the haversine trigonometric function to accurately calculate short distances between two points on the Earth’s surface (16). If the haversine formula determines the distance between them to be shorter than the vaccination radius, cells are eligible for vaccination. The vaccination radius was set at varying distances- 0, 10, 20, 30, 40, or 50 km- to account for several factors that can influence its selection, which are beyond the scope of this manuscript. These factors include variations in wild boar density and distribution, movement and behavior patterns, the velocity of disease spread, natural or man-made barriers, bait distribution logistics, and other practical considerations.

##### 2.2.2.2 Vaccination probability

Once the cell is considered as a neighbor of a vaccinated cell, we calculate its vaccination probability $\left(P_{V,i}\right)$ , a parameter which refers to the probability that an animal remains immunized at time i. $P_{V,i}$ is estimated considering an initial vaccine efficacy and its variations in time, allowing for an immunity decay ( $T_{r})$ from 24 weeks onwards and for vaccination efficiency (see Supplementary material).

We propose a field adaptation vaccination rate $\;(R_{F})$ as a modulator of laboratory-tested vaccine efficacy, allowing the evaluation of different cases of vaccination success depending on the adaptation capacity of the vaccine once it has been applied in the field. The values selected for $R_{F}$ are explained in the Supplementary material.

During each iteration, the vaccination probability is adjusted based on the number of weeks a cell’s neighborhood has been vaccinated, following Equation 1:


(1) $$P_{V,i}=\varepsilon_{i}\;R_{F},$$

with,


$$\varepsilon_{i}=\{\begin{array}{cc} \varepsilon_{0} & \text{If}\;i<T_{e}\\ \varepsilon_{i-1}-\frac{\varepsilon_{i-1}-\varepsilon_{r}}{T_{r}+T_{e}} & \text{If}\;i\geq T_{e} \end{array}$$

where $\varepsilon_{0}$ represents the initial vaccine efficacy, $\varepsilon_{r}$ the reduced vaccine efficacy, $T_{r}$ the number of weeks over which the vaccine efficacy decreases until it reaches the 50% reduced efficacy, $T_{e}$ the number of weeks between vaccination and the point at which efficacy begins to decrease, and $\varepsilon_{i}$ the efficacy in the i-th week (see Supplementary material).

##### 2.2.2.3 Response and immunization time

Vaccination is often not immediate once an infected cell has been identified. In addition, the immune response takes some time to develop (17, 18). We therefore created the variable “response and immunization time (RIT)” to consider both periods. So, we established that unvaccinated cells would transition to a vaccinated state once the number of weeks since the infected neighbor’s outbreak exceeded the RIT variable. We analyzed two different vaccination scenarios, each determined by the estimated start time of the RIT value:

1. An “ideal” scenario, where immunization occurs immediately (i.e., 0 weeks of RIT, with vaccination rate established at 100%, within the entire vaccination radius). This scenario was included for comparison purposes only.
2. A “realistic” scenario, by doubling the vaccination period considered in Barasona et al. (17), i.e., an 8-week delay from the time an outbreak is reported until the population within the vaccination area is considered immunized according to radius and vaccination rate, which is accordance with the immunity development time of 60 days modeled in Martinez-Avilés et al. (18). In total, the model was iterated more than 90 times, with 30 iterations for 8-week RIT, using all possible combinations of vaccination rate and vaccination radius previously mentioned.

We considered that the duration of a vaccination campaign (maximum vaccination period) would be of 12 weeks based on the duration of each CSF vaccination campaign (12).

#### 2.2.3 From vaccinated to infected

For a vaccinated cell to transition to the infected state, they must first revert to the unvaccinated state. A random threshold value is generated: if this value is higher than the efficacy in the ith week ( $\epsilon_{i}$ ), the cell remains in the vaccinated state. However, if the threshold is lower, the cell transitions to the unvaccinated state. For cells that were vaccinated prior to infection, we considered there was a 30% probability of reinfection. This probability of infection is based on the experimental and modeled data showing that animals which survived an attenuated ASF infection could become infectious again (18). This figure reflects a concern regarding vaccine safety, a characteristic that researchers are actively working to improve (see Supplementary material).

### 2.3 Estimation of restriction zones

Following a confirmed disease outbreak, affected EU Member States define restricted areas around the outbreak. Zone III presents the highest level of risk, with domestic pigs affected by ASF. Zone II is an infected zone where only wild boar are affected, and zone I is a disease-free zone bordering zone II and III (38). The demarcation of restriction zones (RZ) changes according to the epidemiological situation and these zones impose movement limitations or bans on certain animals or products, along with other disease control measures, to prevent the spread of a disease into unrestricted areas.

In this study, only ASF cases in wild boar were modeled (thus excluding RZIII, as domestic pigs follow different transmission dynamics). To approximate the control strategy in wild boar, we defined a new CA in which the cellular space was represented by a municipality within the study area, which includes administrative units from Emilia-Romagna, Liguria, Lombardy, Piedmont, and Tuscany Regions—a total of 3,251 municipalities. To simplify the calculation, the number of municipalities was reduced by constructing a polygon that encompasses all municipalities with reported ASF cases during the study period, along with those sharing borders. This reduction resulted in 597 municipalities being considered, of which 293 experienced wild boar outbreaks. In this new cellular model, neighboring municipalities are those that share borders, enabling the identification of the first neighbors of each municipality.

**Figure 2:**
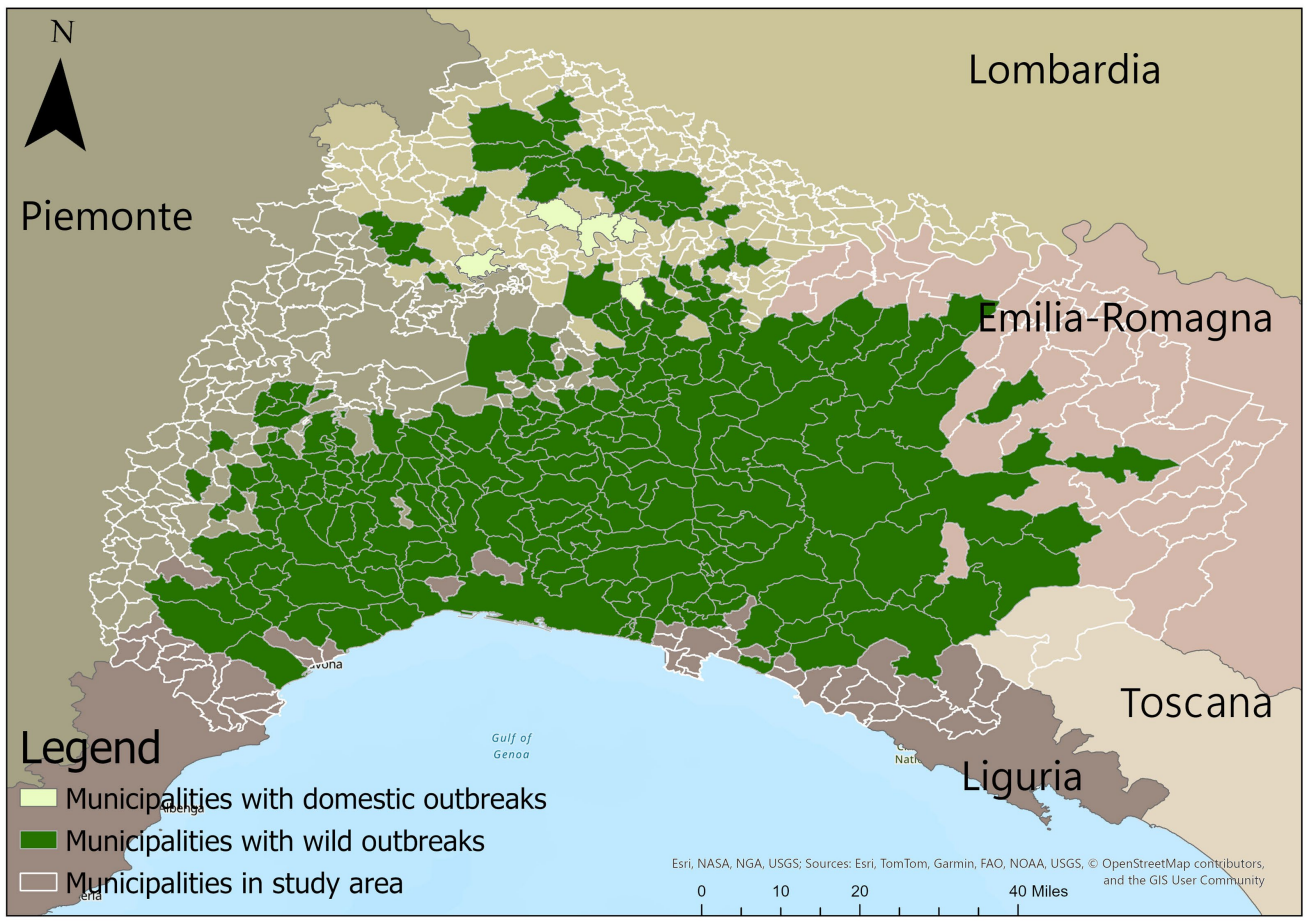
The figure presents a map of the municipalities included in the study area, as they fall within the convex polygon defined by the outbreak coordinates. Dark green highlights the municipalities where wild outbreaks have occurred, while light green marks those with domestic outbreaks. Additionally, municipalities that are either within the study area or are part of the municipalities in the study area are depicted with transparent shading and clearly delineated boundaries.

The states of the municipalities are classified as “*FREE*,” “*RZ2*,” and “*RZ1*,” where “*FREE*” refers to municipalities free of ASF and not sharing a border with any municipality with reported outbreaks, “*RZ2*” designates municipalities with outbreaks in wild boar, and “*RZ1*” corresponds to municipalities bordering those with reported outbreaks. The modeled restriction zones are numbered in Arabic numerals to differentiate them from the Roman numerals used by authorities to list these areas. Figure 2 shows on a map the municipalities that are part of the case study, including those in which an outbreak has been reported.

The iteration algorithm begins with a weekly analysis of infected cells (wild boar outbreaks) from the previous CA vaccination simulation model, assigning the number of infected wild boars to each municipality. When a municipality has more than one infected wild boar, it transitions to RZ2 and all neighboring municipalities in a free state transition to RZ1.

**Figure 3:**
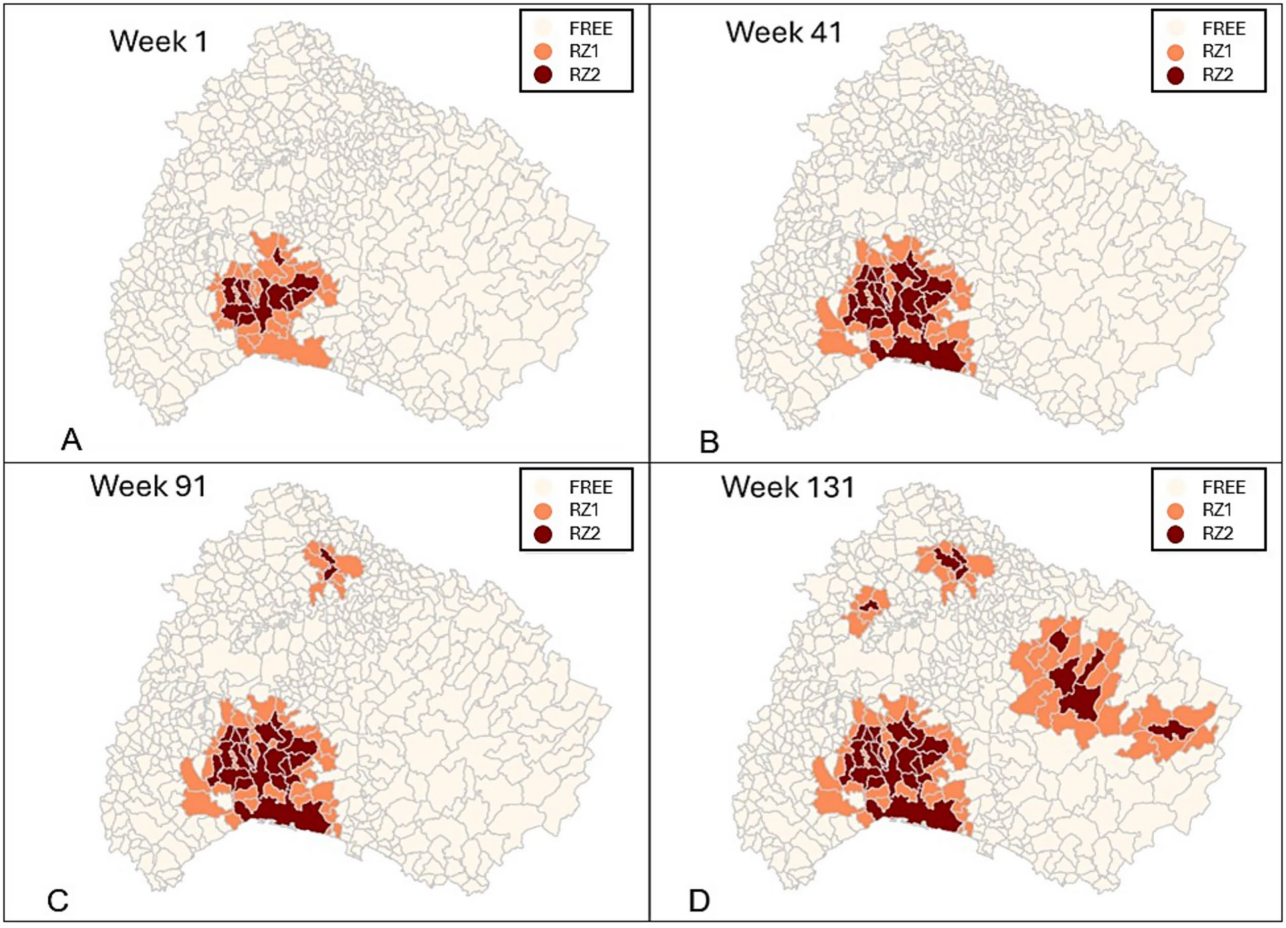
The figure illustrates four time points of the simulation, **(A)** week 1, **(B)** week 41, **(C)** week 91, and **(D)** week 131 by incorporating the following parameters: an initial efficacy of 92%, a field adaptation rate (*R_F_*) of 25%, and a vaccination radius of 30 km and a “response and immunization time (RIT) of 8 weeks and a reduced efficacy of 50% lasting for 10 weeks, followed by a time decay period of 24 weeks.

Municipalities in RZ1 can either transition to RZ2 if an infected municipality is found within their borders in subsequent weeks or remain in RZ1. So far, in the Northern Italy epidemic at the study period, the RZ have not reverted to free zones so the model captures that reality as well, and neither RZ1 nor RZ2 transition to a free state in the simulation. Figure 3 shows on different maps the situation of the municipalities for different time steps according to the zoning for the CA model run.

### 2.4 Estimation of vaccination economic benefits

We estimated the vaccination economic benefits by calculating the losses that would be avoided if vaccination had taken place across the study area.

**Figure 4:**
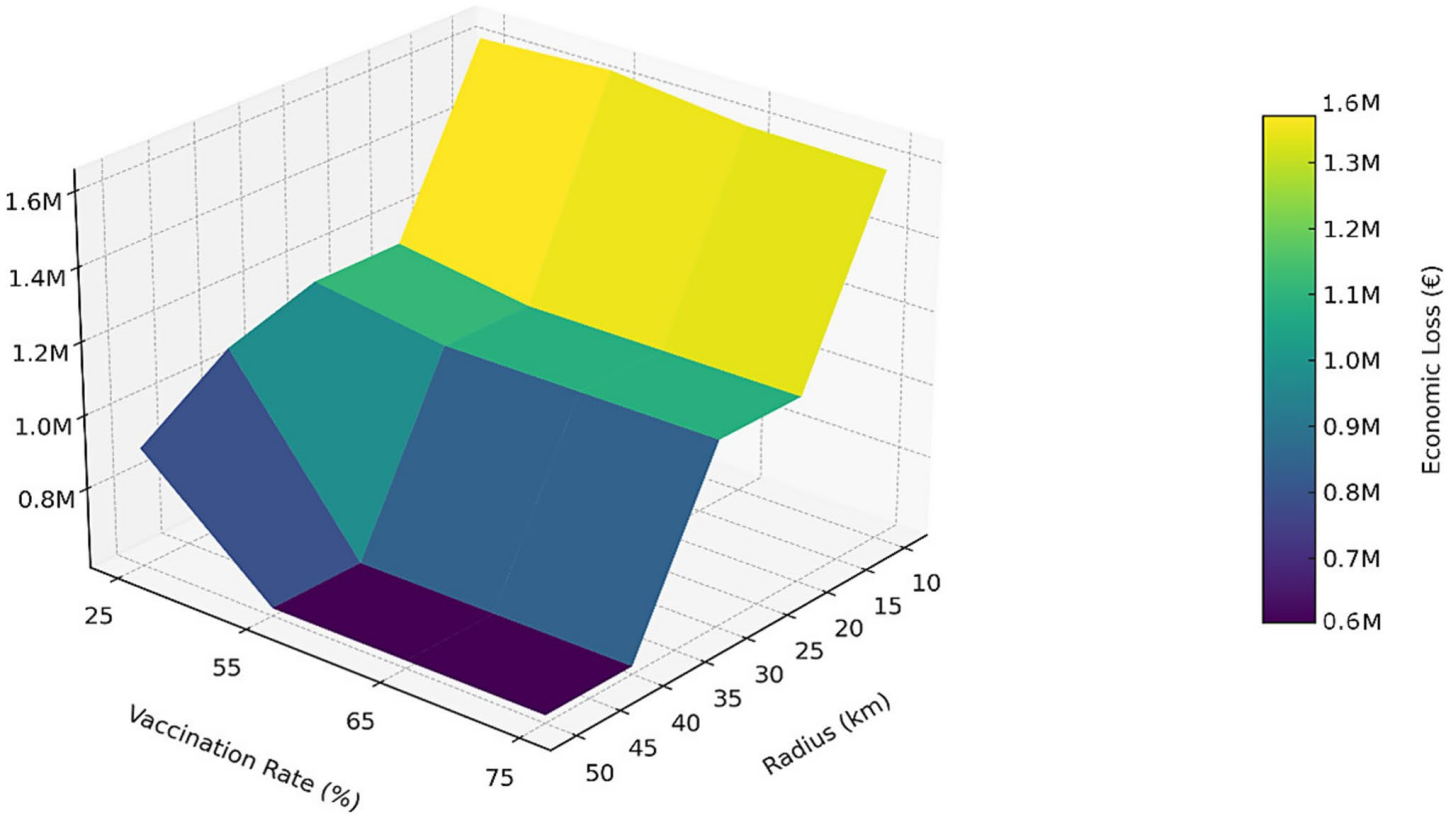
The figure presents a three-dimensional representation of the estimated economic losses (in euros) associated with different combinations of vaccination radius (X-axis) and vaccination coverage rates (Y-axis) during an eight-week campaign. The values are derived from Table 2 and reveal a decreasing trend in losses as the vaccination radius expands and coverage increases—particularly evident for radii greater than 30 km and coverage levels above 55%.

In our simulation, we assumed that the price of pigs and pork products in RZ1 would remain the same as in the free zone, based on the assumption that the Competent Authority would allow movements within the same or other RZ, or within Italy. Only the international trade would be affected. However, since we did not have information about which pig farms were engaged in export, we assumed that exports would be redirected to the national market (Figure 4).

For farms in RZ2, we estimated losses at the municipality level based on the duration that each municipality remained classified as RZ2. We grouped farms within each municipality according to the monetary value of the main product produced (Table 1). Only breeding and fattening pig farms were selected, with market price per kg of live weight. For modeling purposes, we assumed that farms with a capacity for 50 pigs or fewer and breeding farms with closed-cycle operations produced specialized pork products, typically sold at a higher market price than unspecialized pork meat. This reflects common marketing practices among small-scale and short-chain producers—such as direct sales or artisanal processing—who often receive higher market prices than commodity pork, despite some variability across contexts. While this may not apply to all such farms, it reflects the common marketing strategies of small-scale and closed-cycle producers in this area of Italy (IZSUM, personal communication).

**Table 1:** Groups of farms and variables for the calculation of the economic benefit.

Type	Orientation	Type cycle	Specialized	Price (euros/kg)^1^	Weight (kg)	Losses (%)
Farm	Fattening	–	Yes	4	150	50
Farm	Fattening	–	No	2,2	150	30
Farm	Breeding	Closed	Yes	4	150	50
Farm	Breeding	Open	No	0,5	20	30

^1^Available at: https://www.sivempveneto.it/la-peste-suina-fa-calare-i-prezzi-della-carne-di-suino-italiana-lavanzare-dei-contagi-preoccupa-anche-le-imprese-dei-salumi/.

For each group of farms within a municipality, we calculated the lost revenue (no profit) at the municipality level ( NP ), with the following Equation 2:


(2) $$\mathrm{NP}=\frac{C}{M}\cdot Z_{2}\cdot W\cdot P_{F}\cdot R\;$$

Where C represents capacity, M represents the number of study months, $Z_{2}$ represents the number of months in RZ2, W represents the average weight of a pig, $P_{F}$ represents the average market price of meat (unrestricted price), and R represents the reduction coefficient or losses for RZ2. Once the NP value is calculated for each farm—based on the number of weeks spent in RZ2—these individual results are summed to obtain the total NP for the entire study area. This procedure is repeated for each scenario, and the totals are then summed to produce the final overall loss.

The impact of vaccination was assessed using the metric *epidemic reduction (%)*. This metric enables meaningful comparisons of the number of municipalities under restrictions that could avoid entering RZ2 through wild boar vaccination. It is defined as the proportion of municipalities spared from trade restrictions due to infection—i.e., those that remain outside RZ2 when vaccination is applied—compared to the no-vaccination scenario (Table 2).

**Table 2:** Economic losses in euros (€) and epidemic reduction (%) under a vaccination campaign of 8 weeks defined by radius and vaccination field adaptation rate (*R_F_*).

	Vaccination field adaptation rate (*R_F_*)							
	25%		55%		65%		75%	
Radius (km)	Epidemic reduction (%)	Economic losses (€)	Epidemic reduction (%)	Economic losses (€)	Epidemic reduction (%)	Economic losses (€)	Epidemic reduction (%)	Economic losses (€)
10	52.92	1,617,271	59.58	1,634,637	62.08	1,592,962	61.67	1,586,575
20	78.75	1,142,505	83.75	1,087,575	85.00	1,087,575	85.00	1,087,575
30	81.25	1,142,505	84.58	1,087,575	85.00	1,087,575	85.83	1,087,575
40	88.33	1,067,508	88.33	601,800	89.58	601,800	89.17	601,800
50	90.42	905,671	90.83	601,800	90.83	601,800	90.83	601,800

### 2.5 Scenario settings

We evaluated three distinct vaccination scenarios:

- *Non-vaccination scenario:* This represents the situation where no vaccination is implemented, reflecting the current real-world baseline.
- *“Ideal” scenario:* This unrealistic scenario was tested to be able to compare more realistic simulations with “ideal” parameters, such as immediate wild boar vaccination after the first ASF + detection or a 100% vaccination rate. Although unattainable in practice, this idealized framework provides a reference point against which the epidemiological and economic performance of other scenarios can be evaluated.
- *“Realistic” scenarios:* Vaccination immunization was tested with an 8-week delay, using various combinations of vaccination radius (10, 20, 30, 40, and 50 km) and vaccination rate (25, 55, 65, and 75%).

## 3 Results

In this study, we analyzed the impact of varying vaccination parameters on epidemic spread and economic outcomes within a simulated environment. Across all iterations, certain parameters were kept constant, including initial efficacy (92%), vaccination duration (12 weeks), immunity decay (50%), start of the vaccine efficacy reduction period (24 weeks), and duration of the immunity decay until a 50% vaccine efficacy is reached (24 weeks).

### 3.1 Non-vaccination scenario

In the absence of vaccination, no preventive measures were applied. Consequently, by the end of the simulation all 2,130 cells (representing real cases in wild boar) became infected. At the administrative level, all 240 municipalities remained classified under restriction zone 2, replicating the real-world baseline. In this scenario, the estimated losses amounted to €2,131,998.

### 3.2 Ideal vaccination scenario

In the “ideal” vaccination case, nearly all cells were successfully vaccinated except the index case. At the municipal level, most areas remained free of restrictions, with only the index case’s municipality and its immediate neighbors transitioning to restriction zone 1 or 2. Since epidemic-related losses cannot be reduced to zero—losses inevitably occur during the initial outbreak stages—the minimum economic loss reached €601,800. This represents a 71.77% reduction in losses compared to the non-vaccination scenario. In this scenario, epidemic reduction reaches a maximum of 97%, meaning that only seven municipalities remain in RZ2 under the ideal vaccination scenario.

### 3.3 Realistic vaccination scenario

When the vaccination campaign was extended to 8 weeks, results were consistently worse than in the ideal vaccination scenario and markedly better than in the absence of vaccination, illustrating the detrimental effect of delayed implementation.

Table 2 provides a numerical summary of these results, showing epidemic reduction (%) and economic losses (€) for different combinations of vaccination radius and field adaptation rates. Figure 4 presents a three-dimensional visualization of the same data, with the X-axis representing vaccination rate (%), the Y-axis showing vaccination radius (km), and the Z-axis indicating economic loss (€), while the color gradient also reflects economic losses from higher (yellow) to lower (purple) values. Table 2 was constructed from the simulation outputs averaged over the tested scenarios, and Figure 4 derives directly from those numerical results to facilitate visual interpretation of trends.

1. Vaccination rate effect. At a 25% rate, epidemic reduction ranged from 52.92% at 10 km with losses of €1.62 million to 90.42% at 50 km with losses reduced to €905,671. These outcomes remain far worse than the €601,800 observed in the ideal case but clearly superior to the €2.13 million of the non-vaccination scenario. At a 55% rate, protection improved, with 59.58% reduction at 10 km (€1.63 million) and up to 90.83% at 50 km (€601,800). At 65% vaccination rate, results followed a similar gradient: 62.08% at 10 km (€1.59 million) compared to 90.83% at 50 km (€601,800). Finally, at 75% vaccination rate, protection ranged from 61.67% at 10 km (€1.59 million) to 90.83% at 50 km, again stabilizing losses at €601,800.
2. As the vaccination field adaptation rate increased from 25 to 75%, the proportion of municipalities spared from restrictions rose progressively, while economic losses decreased accordingly. However, the improvement was modest at short radii (10 km), where losses consistently exceeded €1.58 million, and became most evident at wider radii (40–50 km), where vaccination rates of 55% or higher consistently brought losses down to €601,800, approaching the benchmark of the ideal scenario.
3. Radius effect. Increasing the vaccination radius consistently mitigated the negative impact of delayed campaigns, although it never reproduced the ideal benchmark. At small radii (10 km), losses exceeded €1.58 million regardless of the vaccination rate, whereas at wider radii (40–50 km), vaccination rate levels ≥55% brought epidemic reduction above 88% and minimized losses to €601,800. Thus, wider radii shifted results closer to the ideal.

The 8-week delay reduced the number of animals protected at the critical onset of epidemic spread. For example, with moderate vaccination rates (55–65%) and radii ≤30 km, losses consistently exceeded €1.08 million, and municipalities protected remained below 85%, a marked deterioration compared with the ideal case, though substantially better than the complete epidemic spread of the anti-ideal.

At a low vaccination rate and short radii (25% at 10 km), the campaign resulted in only 52.92% reduction in restricted municipalities and losses of €1.62 million, a sharp contrast to the near-complete protection of the ideal case but still far from the 100% infection of the anti-ideal. Conversely, at higher vaccination rates (65–75%) with wider radii (40–50 km), the percentage of infected municipalities decreases by 89–91%, worse than the ≥95% achieved under the ideal scenario, yet markedly better than the absence of vaccination.

Best-performing configurations: Even under an 8-week delay, some parameter combinations successfully limited losses to €601,800. These include 55, 65, and 75% vaccination rates combined with 40–50 km radii, as well as 25% vaccination rate at 50 km. Although these outcomes approach the economic efficiency of the ideal scenario, they never match its superior epidemiological impact. Furthermore, the maximum epidemic reduction (97.0%) remained exclusively linked to shorter campaigns, confirming the penalty imposed by delayed implementation.

These findings confirm that both vaccination rate and radius strongly shape outcomes, but timeliness is decisive. An 8-week delay produces results consistently between the ideal benchmark and the anti-ideal scenario: far superior to no vaccination, yet always inferior to immediate deployment. Wider radii partially compensates for delays but cannot fully eliminate their disadvantages. Thus, the ideal scenario serves as a reference point highlighting the benefits of early action, while the anti-ideal underscores the cost of inaction.

## 4 Discussion

The WiBISS (Wild Boar Immunization Strategy Simulator) model represents an innovative tool for evaluating the economic feasibility of vaccination campaigns against ASF in wild boar populations. Unlike traditional epidemiological models focused on transmission dynamics (18–21), WiBISS prioritizes direct economic evaluation, enabling rapid estimation of losses under different intervention scenarios. Similar approaches estimating the economic impact of livestock diseases have been applied in other contexts (22–25). The WiBISS framework, centered on economic efficiency, proves particularly useful in operational contexts where decision-making must be agile and based on limited accessible data (26) (Podgorski et al., 2018).

Nevertheless, despite progress in modeling approaches, their practical applicability is constrained by the absence of a safe and effective vaccine for wild boar. One of the main reasons for this unavailability is the absence of authorized field trials, which are essential to confirm vaccine safety under natural conditions. Although experimental studies have demonstrated vaccine candidates to be both effective and safe (17, 27), additional safety testing—particularly in field settings—remains necessary before large-scale implementation can be considered. Attempts to use ASF vaccines have already been made in Asia, for example in Vietnam, where experimental application in domestic pigs has shown some promising results but also reproductive failure and detection of vaccine-like variants detected in non-vaccinated breeding herds (28). Importantly, in 2025 the WOAH adopted the first internationally agreed standards for ASF vaccines, defining technical requirements for their production and evaluation^4^. These standards represent a milestone toward harmonizing vaccine development and ensuring product quality, safety, and efficacy. However, they primarily address the characteristics of the vaccine itself, while the field application of vaccination—especially in wild boar—still raises specific challenges related to biosafety, logistics, and surveillance. If the benefits of vaccinating wild boar were shown to outweigh the costs of field safety trials, emergency vaccination could be considered as a pragmatic option. However, the risk of long-term carriers—historically described in endemic settings such as Spain in the 1960s—remains a critical concern (29). Demonstrating the absence of this risk would require long-term field evidence, which significantly delays vaccine deployment. Until such uncertainties are resolved, models such as WiBISS should be regarded primarily as tools for theoretical exploration and decision support, rather than as directly applicable solutions.

The WiBISS model indicates that the target losses of the ideal scenario can also be achieved through alternative combinations, the most resource-efficient being a field adaptation parameter of 55% with a radius of 40 km. However, the relationship between the two variables does not follow any straightforward patterns without a complex analysis. Different combinations may yield diverse outcomes, which should be further explored once the model incorporates a broader set of results. Future analyses applying Big Data techniques could provide deeper insights into these complex interactions.

Although estimated losses remain higher than expected under idealized conditions, the WiBISS model is particularly valuable as an initial planning tool for designing comprehensive vaccination campaigns. By providing a rapid and economically informed approximation of potential outcomes under various intervention scenarios, the model allows decision-makers to prioritize resource allocation efficiently during the initial phase of the outbreak response, serving as a guide in the strategic deployment of more detailed epidemiological models and field data-such as transmission dynamics, host behavior, and vaccine efficacy-to refine and complete the intervention strategy. In this way, WiBISS serves as a basis for integrated, phased vaccination planning that combines economic feasibility with biological and ecological realism (20, 30) (Podgorski et al., 2018).

Based on this knowledge, simulations suggest that, despite the potential benefits of wide vaccination radii, economic losses are unlikely to be completely avoided due to inherent delays in detection and geographic distance in the appearance of the first cases due to introduction of the disease into a territory. This reinforces the importance of incorporating early intervention protocols and models to estimate the probability of introduction of ASF into a territory as a fundamental component of vaccination policy (31).

Since the development of the WiBISS prototype in northern Italy, the number of ASF outbreaks has increased, highlighting the growing urgency of effective control measures—most importantly, the development and deployment of a vaccine. In the meantime, when rapid response is essential in the early phases of an epidemic, and epidemiological data may be scarce or delayed, the simplicity of the WiBISS framework offers a strategic advantage as a decision-support tool (30).

Conclusions from different studies show that oral immunization in wild boar can be effective in reducing the clinical symptoms as well as the number of infected animals in a territory (17, 32). These studies indicate that practical application—such as ensuring sufficient bait density and targeting high-risk areas—can lead to significant reductions in ASF prevalence. It is on this last point that the applicability of tools such as WiBISS can help guide regional authorities and wildlife managers toward cost-effective intervention strategies.

One of WiBISS’s main methodological strengths lies in its structural simplification. By omitting detailed virus transmission modeling, the system reduces dependence on complex epidemiological parameters, thus increasing its applicability in data-scarce regions. This feature contrasts with more detailed stochastic approaches, such as those presented by Dankwa et al. (30), which, while enabling more precise epidemic projections, involve greater computational complexity and stringent calibration requirements. Mechanistic and stochastic models have contributed significantly to understanding the spatiotemporal dynamics of ASF, particularly when detailed data are available on group behavior, carcass persistence, and feeding habits (27). In this context, WiBISS complements the methodological framework by offering a more accessible and rapidly deployable alternative.

The model was specifically designed to analyze disease dynamics in wildlife and does not incorporate domestic pig involvement. This exclusion is due to the significant differences in epidemiology, transmission routes, and economic impacts between wildlife and domestic contexts, which require differentiated modeling approaches. Various studies have modeled ASF transmission and control in domestic pig farms using frameworks such as contact networks and compartmental models that incorporate production, movement, and biosecurity factors (31, 33). Therefore, any future integration of wildlife and domestic systems would require hybrid architecture beyond the scope of this study.

From a spatial perspective, the use of spatially explicit (non-homogeneously distributed) cellular automata in WiBISS is a noteworthy methodological choice, enabling realistic modeling of both disease spread and vaccination coverage. This approach supports fine-scale representation of territorial heterogeneities—such as wild boar distribution, geographic barriers, or hard-to-access areas—which directly influence campaign efficacy. It also allows simulation of diffusion scenarios emerging from the interaction of administrative restrictions and animal behavior, contributing to a dynamic interpretation of spatial decision impacts (34, 35).

Nonetheless, the model presents notable limitations. The absence of a commercially available ASF vaccine in Europe necessitated assumptions, based on prior experiences with classical swine fever, thus introducing uncertainty. Likewise, the assumption of homogeneous coverage does not accurately reflect the behavioral and spatial heterogeneity of wild boars, especially in remote or competitively baited areas (32, 36).

The current capabilities of WiBISS reflect only the initial phase of its development; its adaptability to empirical inputs and digital deployment underscores its potential as a decision-support system for diverse epidemiological contexts. WiBISS provides a novel contribution to ASF management, particularly under data-limited conditions where rapid decision-making is critical. By balancing simplicity with applicability, the model equips stakeholders with an efficient tool for economic evaluation and spatial planning. Its long-term utility will hinge on its ability to incorporate empirical data from ongoing field studies, including key parameters such as bait uptake, degradation rates, and interspecies competition. However, the model currently has certain limitations: it produces results with a single temporal resolution and does not store intermediate results, but only the final results of each run and a set of random intermediate images for scenario visualization. Since our main objective was to compare the overall economic results between different vaccination strategies, the aggregated results at the endpoints provide the most interpretable results. The development of an online interface—currently underway—will further enhance its accessibility, allowing users to input context-specific data and simulate customized intervention scenarios. Through continued refinement and potential integration with domestic pig transmission models, WiBISS could evolve into a reference platform for pig producers, veterinary authorities, and policy makers engaged in controlling ASF across diverse epidemiological landscapes (31, 33, 35, 39).

## Data Availability

The data analyzed in this study is subject to the following licenses/restrictions: There are datasets that belong to the Italian government; others are public. Requests to access these datasets should be directed to WOAH (for WAHIS data), IZSUM for Italian data.

## Footnotes

^1^ https://wahis.woah.org/#/event-management

^2^ https://vacdiva.eu/

^3^ https://www.sivempveneto.it/la-peste-suina-fa-calare-i-prezzi-della-carne-di-suino-italiana-lavanzare-dei-contagi-preoccupa-anche-le-imprese-dei-salumi/

^4^ https://www.woah.org/en/article/african-swine-fever-woah-vaccine-standard-adopted/
