# Supplementary material for: WiBISS: a tool to estimate avoided lost revenue of African swine fever wild boar vaccination at municipality level
Source: Front Vet Sci. 2025 Oct 24;12:1667173. doi: 10.3389/fvets.2025.1667173 (PMC12591982; doi:10.3389/fvets.2025.1667173)
Supplement: Supplementary file 1 [file Data_Sheet_1.PDF]

Supplementary Material

1 Glossary of terms

Vaccine efficacy: The protective capacity of a vaccine as measured in vaccination/challenge trials. For ASF, we assumed a vaccine efficacy of 92% based on the experimental results in wild boar (Barasona et al., 2019).

Vaccine safety: The extent to which a vaccine prevents transmission to naïve animals. Safety trials of ASF vaccine candidates in wild boar are ongoing, but modeling studies suggest that vaccinated animals could become infectious again (Martínez Avilés et al., 2023) so we considered this possibility if immunity decayed beyond 50%.

Vaccination effectiveness: The degree to which vaccination induces protective immunity at the population-level. For ASF, no field data are available in Europe; estimates are based on classical swine fever vaccination in wild boar. Field studies showed seroprevalence up to 60% , with seroconversion rates of 37-72% and modeling suggested 40–60% population immunity could halt spread (Rossi et al., 2015; Moennig, 2015). For modeling, a **vaccination effectiveness rate** of 55% was assumed, with scenarios of 25%, 65% and 75%.

Immunity decay: The decline of protection over time after vaccination. Experimental studies in wild boar showed protection lasting at least 54 days, while antibodies persisted >3 months but were not always protective (Barasona et al. (2019, 2021); Nurmoja et al, 2017). In domestic pigs, immunity declined after 130-170 days, and with AVAC ASF LIVE full protection lasted 150 days (5 months) but survival dropped to 40% at 180 dpv (Sánchez-Cordón et al. 2020; Lotonin et al.,2025, Van Diep et al., 2025). For modeling, immunity was assumed to wane after 6 months (24 weeks), with a 50% reduction over the following 6 months.
